# The Use of Saliva in the Diagnosis of Oral and Systemic Diseases

**DOI:** 10.1155/2019/9149503

**Published:** 2019-06-09

**Authors:** Anna Zalewska, Napoleon Waszkiewicz, Rosa María López-Pintor

**Affiliations:** ^1^Conservative Dentistry, Medical University in Bialystok, Poland; ^2^Department of Psychiatry, Medical University of Bialystok, Poland; ^3^Department of Dental Clinical Specialties, School of Dentistry, Complutense University, Madrid, Spain

Saliva produced by the salivary glands plays the most important role in oral homeostasis, including cleaning and moisturizing both oral mucosa and teeth, facilitating articulation and swallowing. Saliva determines the protection of the surface of the teeth and the mucous membranes of the oral cavity against biological, chemical, and mechanical insults [1]. Saliva may be considered as a major component of the oral host defenses, which constitute a first line of defense against ROS-induced agents in tobacco smoke, alcohol, drugs, and other xenobiotics of the diet [2]. As a result of rapid development of salivaomics, saliva is also recognized as a pool of biomarkers. Whole saliva is a good noninvasive diagnostic material that could be a substitute for blood in the monitoring, prognosis, and treatment of many general diseases. Interest in saliva is not surprising because saliva contains a wide range of ingredients that reflect the level of biomarkers in real time as well as the composition of the plasma. What is more, saliva biomarkers cover changes in the biochemical indicators of RNA, DNA, and proteins of oral microbiota.

As we enter the era of genomic medicine, we think that sialochemistry will replace the biochemical analysis of blood in everyday medical clinical practice. Saliva offers many advantages: easy and noninvasive collection, with no risk of needlestick injuries, and a good cooperation of the patients. Moreover, saliva compounds are characterized by a relatively long shelf life compared to blood [3] and its collection may provide a cost-effective approach for the screening of large population and eliminate the risk of contracting infectious diseases for the doctor and patients.

This special issue includes high quality and original research papers showing easily accessible salivary markers in the diagnosis, monitoring, and progression of the systemic diseases.

The review of A. Roi et al. summarizes the latest researches in saliva-related studies and explores the information and correlations that saliva can offer regarding the systemic and oral diseases, highlighting its great potential of diagnosis.

A. I. Lorenzo-Pouso et al. described overall perspective of salivary biomarkers identified in several oral diseases by means of molecular biology approaches.

A. Kułak-Bejda et al. proved that saliva could be recommended as an excellent material for biochemical, toxicological, and immunological diagnostics of not only oral cavity or systemic diseases but also in the still unexplored field of neuropsychiatry.

The study of R. Koshi et al. demonstrated that the lactoferrin and *α*1-antitrypsin in gingival fluid was positively related to the severity of periodontal status. The authors claimed that the measurements of these biomarkers could be applied to periodontal clinical practice.

C. Labat et al. identified salivary phosphate as an independent predictor of carotid artery intima media thickness and the association of several salivary electrolytes with the heart rate. The authors claimed that the differential association of salivary electrolytes with cardiovascular phenotypes indicates that these electrolytes should be further studied for their predictive value as noninvasive biomarkers for determining cardiovascular risk.

S. Alassiri et al. described recently developed practical, convenient, inexpensive, noninvasive, and quantitative mouthrinse and PISF (peri-implant sulcular fluid)/GCF (gingival crevicular fluid)/PoC (point-of-care)/chair-side lateral-flow aMMP-8 immunoassays (PerioSafe and ImplantSafe/OralLyser) to detect, predict, and monitor successfully the course, treatment, and prevention of periodontitis and peri-implantitis, respectively.
